# Over a third of palliative medicine physicians meet burnout criteria: Results from a survey study during the COVID-19 pandemic

**DOI:** 10.1177/02692163231153067

**Published:** 2023-02-15

**Authors:** Jason W Boland, Monisha Kabir, Edward G Spilg, Colleen Webber, Shirley H Bush, Fliss Murtagh, Peter G Lawlor

**Affiliations:** 1 Wolfson Palliative Care Research Centre, Hull York Medical School, University of Hull, UK; 2 Hull York Medical School, University of York, UK; 3 Bruyère Research Institute, Ottawa, ON, Canada; 4 Division of Geriatric Medicine, Department of Medicine, University of Ottawa, Ottawa, ON, Canada; 5 Ottawa Hospital Research Institute, Ottawa, ON, Canada; 6 Division of Palliative Care, Department of Medicine, University of Ottawa, Ottawa, ON, Canada; 7 Clinical Epidemiology Program, Ottawa Hospital Research Institute, Ottawa, ON, Canada; 8 Bruyère Continuing Care, Ottawa, ON, Canada

**Keywords:** Burnout, psychological, resilience, depression, alcohol, palliative medicine, physicians, COVID-19, surveys and questionnaires

## Abstract

**Background:**

Palliative medicine physicians may be at higher risk of burnout due to increased stressors and compromised resilience during the COVID-19 pandemic. Burnout prevalence and factors influencing this among UK and Irish palliative medicine physicians is unknown.

**Aim:**

To determine the prevalence of burnout and the degree of resilience among UK and Irish palliative medicine physicians during the COVID-19 pandemic, and associated factors.

**Design:**

Online survey using validated assessment scales assessed burnout and resilience: The Maslach Burnout Inventory Human Services Survey for Medical Personnel [MBI-HSS (MP)] and the Connor-Davidson Resilience Scale (CD-RISC). Additional tools assessed depressive symptoms, alcohol use, and quality of life.

**Setting/participants:**

Association of Palliative Medicine of UK and Ireland members actively practising in hospital, hospice or community settings.

**Results:**

There were 544 respondents from the 815 eligible participants (66.8%), 462 provided complete MBI-HSS (MP) data and were analysed. Of those 181/462 (39.2%) met burnout criteria, based on high emotional exhaustion or depersonalisation subscales of the MBI-HSS (MP). A reduced odds of burnout was observed among physicians who worked ⩽20 h/week (vs 31–40 h/week, adjusted odds ratio (aOR) 0.03, 95% confidence interval (CI) 0.002–0.56) and who had a greater perceived level of clinical support (aOR 0.70, 95% CI 0.62–0.80). Physicians with higher levels of depressive symptoms had higher odds of burnout (aOR 18.32, 95% CI 6.75–49.73). Resilience, mean (SD) CD-RISC score, was lower in physicians who met burnout criteria compared to those who did not (62.6 (11.1) vs 70.0 (11.3); *p* < 0.001).

**Conclusions:**

Over one-third of palliative medicine physicians meet burnout criteria. The provision of enhanced organisational and colleague support is paramount in both the current and future pandemics.

**What is already known about the topic?**

- Burnout among physicians is associated with decreased physician wellbeing, reduced quality of patient care, and increased workforce issues
- Reported rates of burnout have varied among previous surveys of palliative medicine physicians across different countries and settings
- Resilience is protective against burnout

**What this paper adds?**

- The first reported baseline population prevalence estimate of burnout among UK and Irish palliative medicine physicians, which should be interpreted in the context of the COVID-19 pandemic
- This survey found a 39.2% prevalence of burnout palliative medicine physician respondents during the COVID-19 pandemic. This finding is within the range (31-76%) reported for physicians in general during the pandemic, but higher than previously reported pre-pandemic estimates in palliative medicine physicians in other high-income countries (15%).
- Perception of greater support from colleagues for palliative medicine physicians is associated with reduced burnout

**Implications for practice, theory or policy**

- Rather than focusing solely on individual level interventions, systemic causes of physician burnout need to be addressed.
- There is a need for enhanced colleague and organisational support to reduce burnout and its potential effect on physician wellbeing, patient care and the workforce.
- Having determined baseline prevalence estimates, ongoing levels of burnout and resilience need to be monitored.

## Introduction

Burnout has been characterised by Maslach et al. as ‘a state of emotional exhaustion, depersonalisation, and diminished sense of personal accomplishment and self-achievement as an outcome of extensive job-related stress’.^1,2^ Among healthcare professionals, burnout is associated with poorer mental and physical health,^3^ decreased quality of patient care,^4–6^ increased medical errors,^7,8^ less empathy,^9,10^ increased work absences, and poorer staff retention.^11^

Psychological resilience is the ability to cope mentally or emotionally with a crisis or to return to pre-crisis status quickly.^12^ Resilience enables people to adapt and flourish in adverse situations.^13^ Higher resilience can protect against burnout.^14^ Interventions to increase resilience can be helpful as part of a skill-building approach to burnout and stress reduction in clinicians.^15^

Internationally, physician burnout is under-recognised and under-reported.^16^ It has been suggested that anyone working close to human suffering will develop some aspects of burnout at some point in their career.^17^ Prior to the pandemic, a systematic review reported the prevalence of burnout in palliative medicine physicians as 15% across seven high-income countries.^18^ During the COVID-19 pandemic, physicians (among other healthcare providers) around the world have reported higher levels of burnout, ranging from 31% to 76%.^19–22^ In Canada, the incidence rate of physician visits to psychiatry and primary care providers for mental health and substance use related issues increased on average by 13% per physician during the pandemic.^23^

Although burnout reportedly occurs frequently in palliative medicine physicians,^24–33^ there are no published data on levels of burnout and resilience in United Kingdom (UK) and Irish palliative medicine physicians. There are also limited international data on burnout and resilience in palliative medicine physicians during the COVID-19 pandemic. Therefore, the aim of this study was to determine the prevalence of burnout and the degree of resilience among UK and Irish palliative medicine physicians, and to examine associated factors, within the context of the COVID-19 pandemic.

## Methods

We conducted an online cross-sectional survey study of experiences of burnout and resilience among palliative medicine physicians in the UK and Ireland. Data collection was completed via a voluntary online survey distributed between December 4, 2020 and April 23, 2021, thus including the second wave of the COVID-19 pandemic in both countries. The Checklist for Reporting Results of Internet E-Surveys (CHERRIES) was used to report our results.^34^ This study received approval from a local research ethics board (Hull York Medical School Ethics Approval, REF 20 55, 30th November 2020).

### Participants

Study participants were recruited from the membership of the Association for Palliative Medicine of UK and Ireland, the largest organisation of specialist palliative medicine physicians across the UK and Ireland, with 815 ‘full members’. These are palliative medicine physicians (specialist trainees, associate specialists, and consultants) in current practice with full Association for Palliative Medicine membership status and who were eligible to participate. There was no restriction on setting(s) of work. No incentives were offered to respondents.

### Survey development

The research team, assisted by experts in palliative medicine, designed the online survey based on a review of the literature and a previously published pre-pandemic Canadian survey .^24^ The survey was further modified based on pilot testing among palliative medicine specialists in the UK (*n* = 6) to ensure comprehension and ease of use.

The final survey (Supplemental File 1) consisted of 54 questions distributed over 19 online pages with an estimated completion time of 10 min. Items were not randomised or alternated, and adaptive questioning was not used. Skip logic was used to facilitate answers about practice setting and related questions. Respondents were able to go back to change their answers. The number of questionnaire items per page varied, but was optimised to reduce the need for scrolling.

### Distribution

The survey was administered online via a secure online platform, SurveyMonkey^®^ (www.surveymonkey.com/). On December 4, 2020, the survey invitation with weblink was emailed by the Association of Palliative Medicine administrative assistant to its full members (*n* = 815). Two follow-up reminder emails were sent out on December 18, 2020 and February 10, 2021. The anonymous survey could only be completed once by each participant.

The survey invitation included details of the study purpose, names of study investigators, details of ethical approval, and the anticipated time for survey completion. Respondents provided informed written consent electronically prior to participating.

### Data collection

Information collected via the survey included physician demographic and practice characteristics, level of formal supervision in palliative medicine (i.e. dedicated time for supervisory support/development), self-perceived level of support from others at practice site(s), role, validated tools assessing burnout and resilience, as well as depressive symptoms, alcohol consumption, and quality of life.

### Outcome measures

The Maslach et al. Burnout Inventory Human Services Survey for Medical Personnel [MBI-HSS (MP)],^35^ a validated 22-item questionnaire, was used to assess burnout. The MBI-HSS (MP) has three subscales which measure emotional exhaustion, depersonalisation, and personal accomplishment. Each item is rated using a seven-point Likert scale, ranging from never (0) to everyday (6), and item scores are summed per subscale. Subscale cutpoints of ⩾27 for high emotional exhaustion, ⩾10 for high depersonalisation, and ⩽33 for low personal accomplishment were defined based on normative data in medical professionals for risk of burnout.^35,36^ For this study, burnout was primarily defined as present if a participant had high emotional exhaustion or high depersonalisation subscale scores.^4^ This is consistent with other studies measuring physician burnout,^26,37,38^ and assumes that physicians still manage to find fulfilment in their work. Thus, physicians may experience burnout while still displaying a high level of personal accomplishment. Recognising that many definitions of burnout exist,^39^ we also report an alternate burnout prevalence, based on respondents having high emotional exhaustion, high depersonalisation or low personal accomplishment (secondary definition).^40^

The 25-item Connor-Davidson Resilience Scale (CD-RISC)^41^ was used to assess resilience. Respondents indicated the level of agreement with each scale item on a five-point Likert scale ranging from not true at all (0) to true nearly all the time (4). The total score, representing the sum of item scores, was treated as a continuous variable (range: 0–100), with higher total scores indicating greater resilience.

Additional tools were used to assess depressive symptoms, alcohol consumption and quality of life. The Patient Health Questionnaire-2 (PHQ-2)^42^ is a two-item screening tool that assesses the frequency of depressive symptoms and anhedonia. Each item is scored on a scale of 0–3, with total scores ranging from 0 to 6. A score of 3 is the optimal cutpoint, indicating that major depressive disorder is likely.^42^ The Alcohol Use Disorders Identification Test – Consumption (AUDIT-C),^43^ a three-item questionnaire with scores ranging from 0 to 4 for each item, was used to assess alcohol consumption and categorised as low (0–3), moderate (4–5) and high-risk (6–12) drinking. Self-perceived change in alcohol consumption during the COVID-19 pandemic was recorded on a 5-point Likert scale.

### Statistical analysis

We used descriptive statistics, including counts and proportions for categorical variables, means and standard deviations (SDs) for normally distributed continuous variables, and medians and interquartile ranges (IQRs) for skewed continuous variables, to describe respondent characteristics and outcome distributions. We evaluated associations between respondent characteristics and: (i) burnout using chi-square tests of independence, and odds ratios (OR) derived from unadjusted and adjusted logistic regression; and (ii) resilience score using *t*-tests and unadjusted and adjusted linear regression. Selection of independent variables for the multivariable regression models was based on a priori designated clinical importance as determined by the study team. Given their potential correlation with years in medical practice, and consequent collinearity concerns, age and years in palliative medicine practice were not included in these analyses. Data were analysed using SAS version 9.3.

## Results

**Figure 1.**
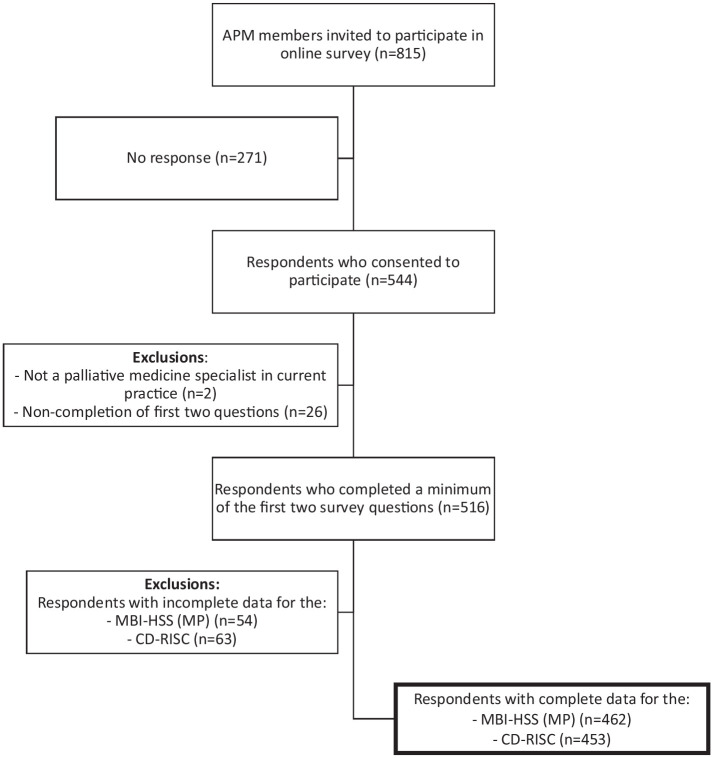
Flow diagram of survey participation. APM: Association of Palliative Medicine; MBI-HSS (MP): Maslach Burnout Inventory Human Services Survey for Medical Personnel; CD-RISC: Connor-Davidson Resilience Scale.

Of 815 invited physicians with full Association of Palliative Medicine membership, 544 (66.8%) consented to participate. Excluding those not in current practice (*n* = 2) or who did not answer the first two demographic questions (*n* = 26), there were 516 respondents. Respondents who completed a minimum of the first two demographic questions were included in the analysis. As not all respondents answered all survey questions, the denominator varies for individual questions. A flow diagram of study participants is presented in Figure 1.

The characteristics of the 516 respondents are summarised in Table 1. The majority (*n* = 487, 94%) of respondents were aged 31–60 years. Most (*n* = 424, 82%) were female and 64% (*n* = 332) were fully trained consultants. The majority (*n* = 443, 86%) of respondents had been in practice for over 10 years, and 85% (*n* = 440) had worked for 5 years or more in palliative medicine. 11% (*n* = 58) screened positive for depression. Pandemic alcohol consumption increased in 31% (*n* = 162); 10% (*n* = 52) were in the high-risk drinking category.

**Table 1.** Respondent characteristics (*n* = 516).

Characteristics	*N* (%)
Age	
21–30	18 (3.5)
31–40	156 (30.2)
41–50	215 (41.7)
51–60	116 (22.5)
61–65	9 (1.7)
>65	2 (0.4)
Sex	
Male	90 (17.4)
Female	424 (82.2)
Prefer not to answer	2 (0.4)
Role^a^	
Consultant in Palliative Medicine	332 (64.3)
Staff grade or Associate Specialist in Palliative Medicine	87 (16.9)
Specialist Trainee or Registrar in Palliative Medicine	80 (15.5)
Other	17 (3.3)
Years in medical practice	
<10 years	73 (14.2)
11–20 years	185 (35.8)
21–30 years	195 (37.8)
>30 years	63 (12.2)
Years in Palliative Medicine	
<1 year	13 (2.5)
1–4 years	63 (12.2)
5–9 years	109 (21.1)
10–20 years	223 (43.2)
>20 years	108 (20.9)
Setting^b^	
Cancer centre	85 (16.5)
Community hospital	62 (12.0)
General hospital	197 (38.2)
Teaching hospital	159 (30.8)
Hospice Inpatient Unit	396 (76.7)
Long-term care/Nursing or Care Home	97 (18.8)
Community	286 (55.4)
Outpatient clinic	243 (47.1)
Other	19 (3.7)
Contracted hours per week in Palliative Medicine	
0–10 h	7 (1.4)
11–20 h	29 (5.6)
21–30 h	151 (29.3)
31–40 h	256 (49.6)
41–50 h	69 (13.4)
51–60 h	4 (0.8)
Actual hours per week in Palliative Medicine	
⩽20 h	19 (3.7)
21–30 h	97 (18.8)
31–40 h	161 (31.2)
41–50 h	166 (32.2)
>50 h	73 (14.1)
Provision of on-call for Palliative Medicine	
Yes	471 (91.3)
No	45 (8.7)
Frequency of on-call for Palliative Medicine	
1 in 7 or less frequently	153 (32.6)
1 in 4–1 in 6	281 (59.8)
1 in 3 or more frequently	36 (7.7)
Formal supervision in Palliative Medicine	
Yes	201 (41.1)
No	288 (58.9)
Perceived level of support from others at clinical sites (as measured by visual analogue scale scored from 1 (no support) to 10 (maximum support))	Median (IQR) 8 (6–9)
Not reported	27 (5.2)
Distribution of workload, according to percent of time spent in:	Median (IQR)
Palliative Medicine clinical work (*n* = 489 responded)	70 (50–80)
Non-Palliative Medicine clinical work (*n* = 288 responded)	0 (0–5)
Non-clinical work (*n* = 477 responded)	20 (15–33)
Other work (*n* = 145 responded)	5 (0–21)
Patient Health Questionnaire-2 (PHQ-2) score	
⩾3	58 (11.2)
<3	394 (76.3)
Not reported	64 (12.4)
Change in alcohol consumption during COVID-19 pandemic	
Decreased a lot	34 (6.6)
Decreased a little	51 (9.9)
Stayed about the same	205 (39.7)
Increased a little	141 (27.3)
Increased a lot	21 (4.1)
Not reported	64 (12.4)
Alcohol Use Disorders Identification Test – Consumption (AUDIT-C) score	
0–3 (low-risk drinking)	253 (49.0)
4–5 (moderate-risk drinking)	131 (25.4)
6–12 (high-risk drinking)	52 (10.1)
Not reported	80 (15.5)

a Consultants: fully trained specialists in palliative medicine (work exclusively in palliative medicine); Staff grade or Associate Specialist in palliative medicine: non-consultant grade specialist in palliative medicine (may also work in non-palliative medicine work, such as primary care/family medicine); Specialist Trainee or Registrar in palliative medicine: physician undergoing palliative medicine speciality training (work exclusively in palliative medicine).

b Respondents could select more than one care setting.

### Burnout

Fifty-four (10.5%) respondents did not complete the MBI-HSS (MP), leaving 462 respondents with complete data to characterise the presence of burnout. Of these, 36.6% (*n* = 169) had high emotional exhaustion, 14.1% (*n* = 65) had high depersonalisation, and 19.9% (*n* = 92) had low personal accomplishment (Table 2). Over one-third (*n* = 181, 39.2%) of respondents met the primary study definition of burnout (high emotional exhaustion or high depersonalisation). A total of 219 respondents (47.4%) met the secondary definition of burnout (high emotional exhaustion or high depersonalisation or low personal accomplishment).

**Table 2.** Maslach Burnout Inventory^a^ scores for 462 respondents with complete data on this tool.

MBI-HSS (MP)^a^ Sub-scale	*N* (%)
Emotional Exhaustion (EE)	
⩽18 (Low)	167 (36.1)
19–26	126 (27.3)
⩾27 (High)	169 (36.6)
Depersonalisation (DP)	
⩾ 5 (Low)	307 (66.4)
6–9	90 (19.5)
⩾10 (High)	65 (14.1)
Personal Accomplishment (PA)	
⩾40 (High)	207 (44.8)
39–34	163 (35.3)
⩽33 (Low)	92 (19.9)
Burnout – using primary burnout definition method (EE ⩾ 27 or DP ⩾ 10)^4^	
Yes	181 (39.2)
No	281 (60.8)
Burnout – using secondary burnout definition method (EE ⩾ 27 or DP ⩾ 10 or PA ⩽ 33)^40^	
Yes	219 (47.4)
No	243 (52.6)

a Maslach Burnout Inventory Human Services Survey for Medical Personnel [MBI-HSS (MP)].

Logistic regression analyses of associations between respondent characteristics and burnout are summarised in Table 3. In the unadjusted analysis, those with staff grade or trainee status, in addition to those with a higher perceived level of support, were less likely to have burnout, whereas absence of formal supervision in palliative medicine, high levels of depressive symptoms, working over 40 h per week, and those with high-risk alcohol consumption were all more likely to have burnout. There were no differences in burnout according to respondent sex, years in palliative medicine, contracted hours per week in palliative medicine, or provision of or frequency of on-call service.

**Table 3.** Bivariate and multivariable logistic regression estimating the odds of burnout, excluding individuals with missing Maslach Burnout Inventory Human Services Survey for Medical Personnel [MBI-HSS (MP)]data for outcome measurement (row %).

	Burnout, *n* (%)	No burnout, *n* (%)	Unadjusted OR (95% CI)	Adjusted OR (95% CI)
Sex				
Male	36 (43.9)	46 (56.1)	1.26 (0.78–2.04)	1.31 (0.70–2.43)
Female	145 (38.4)	233 (61.6)	1.00	1.00
Prefer not to answer	0 (0.0)	2 (100.0)		
Role	133 (44.6)	165 (55.4)	1.00	1.00
Consultant in palliative medicine	25 (31.6)	54 (68.3)	**0.58 (0.34–0.99)^c^**	0.93 (0.47–1.84)
Staff grade or Associate Specialist in palliative medicine	18 (25.3)	53 (74.6)	**0.42 (0.23–0.75)^c^**	0.78 (0.28–2.15)
Specialist Trainee or Registrar in palliative medicine	5 (35.7)		0.68 (0.22–2.09)	0.86 (0.20–3.66)
Other		9 (64.3)		
Years in medical practice				
<10 years	15 (23.8)	48 (76.1)	1.00	1.00
11–20 years	62 (36.7)	107 (63.3)	1.85 (0.96–3.58)	1.02 (0.36–2.91)
21–30 years	75 (43.9)	96 (56.1)	**2.53 (1.31–4.86)^c^**	1.59 (0.53–4.80)
>30 years	29 (49.1)	30 (50.8)	**3.20 (1.47–6.95)^c^**	1.79 (0.51–6.23)
Actual hours per week in palliative medicine				
⩽20 h	2 (10.5)	17 (89.5)	0.23 (0.05–1.04)	**0.03 (0.002–0.56)^c^**
21–30 h	28 (31.8)	60 (68.2)	0.86 (0.49–1.51)	0.73 (0.36–1.47)
31–40 h	51 (35.2)	94 (64.8)	1.00	1.00
41–50 h	69 (46.9)	78 (53.1)	**1.63 (1.02–2.61)^c^**	1.38 (0.77–2.46)
>50 h	31 (49.2)	32 (50.8)	**1.84 (1.01–3.37)^c^**	1.09 (0.52–2.28)
Formal supervision in palliative medicine				
Yes	56 (29.6)	133 (70.4)	1.00	1.00
No	125 (45.8)	148 (54.2)	**2.03 (1.37–3.01)^c^**	1.25 (0.75–2.09)
Perceived level of support from others at clinical sites				
Median (IQR)	7 (5–8)	8 (7–9)	**0.66 (0.59–0.74)^c^**	**0.70 (0.62–0.80)^c^**
Patient Health Questionnaire-2 (PHQ-2)^a^				
<3	124 (31.5)	270 (68.5)	**18.73 (7.84–44.77)^c^**	**18.32 (6.75–49.73)^c^**
⩾3	52 (89.7)	6 (10.3)	1.00	1.00
Alcohol Use Disorders Identification Test – Consumption (AUDIT-C)^b^				
0–3 (low-risk drinking)	88 (34.8)	165 (65.2)	1.00	1.00
4–5 (moderate-risk drinking)	55 (41.9)	76 (58.0)	1.35 (0.87–2.08)	0.99 (0.58–1.69)
6–12 (high-risk drinking)	27 (51.9)	25 (48.1)	**2.01 (1.10–3.68)^c^**	1.54 (0.74–3.22)

OR: odds ratio; CI: confidence interval.

a Individuals with missing PHQ-2 scores excluded from analysis (*n* = 64; with 54 of these also missing MBI-HSS (MP) scores).

b Individuals with missing AUDIT-C scores excluded from analysis (*n* = 80; with 54 of these also missing MBI-HSS (MP) scores).

c Bold entries highlight the statistically significant results.

Two respondents did not respond regarding sex and were excluded. In this adjusted model, working 20 or fewer hours per week compared to 31–40 h per week (aOR 0.03, 95% CI 0.002–0.56) and greater perceived level of support (aOR 0.70, 95% CI 0.62–0.80) were independently associated with a reduced odds of burnout. High level of depressive symptoms was associated with an increased odds of burnout (aOR 18.32, 95% CI 6.75–49.73).

### Resilience

Sixty-three (12%) respondents did not complete the CD-RISC, leaving 453 respondents with complete data to characterise resilience. Although the overall group mean (SD) total CD-RISC score was 67.1 (11.8), respondents who met primary burnout criteria had significantly lower scores than those without burnout: 62.6 (11.1) versus 70.0 (11.3), respectively (*p* < 0.001).

Bivariate and multiple linear regression associations between respondent characteristics and resilience (mean CD-RISC scores), reported as adjusted mean difference (aMD) in the multivariable model are summarised in Table 4. Mean CD-RISC scores were significantly lower for Staff Grade or Associate Specialists in palliative medicine in contrast to consultants (aMD: −3.90, 95% CI −6.84 to −0.96). Compared to respondents who worked 31–40 h a week, respondents who worked 21–30 h per week had lower CD-RISC scores (aMD: −3.35, 95% CI −6.34 to −0.35) while those who worked over 50 h per week had higher CD-RISC scores (aMD: 4.49, 95% CI 1.13 to 7.84). Respondents with depressive symptoms had a CD-RISC score that was 9.74 points lower (95% CI −12.91 to −6.58) than those with no depressive symptoms.

**Table 4.** Bivariate and multivariable linear regression estimating difference in mean Connor-Davidson Resilience Scale (CD-RISC) total score for those with complete CD-RISC data (*n* = 453).

	Mean score (SD)^a^	Unadjusted mean difference	Adjusted mean difference (95% CI)
Sex			
Male	66.6 (11.9)	−0.50 (−3.33, 2.32)	−1.55 (−4.36, 1.25)
Female	67.1 (11.8)	0.00	0.00
Prefer not to answer	79.0 (5.6)		
Role			
Consultant in palliative medicine	68.2 (11.9)	0.00	0.00
Staff grade or Associate Specialist in palliative medicine	64.3 (12.0)	**−3.98 (−6.91, −1.05)^d^**	**−3.90 (−6.84, −0.96)^d^**
Specialist Trainee or Registrar in palliative medicine	66.4 (10.2)	−1.73 (−4.80, 1.35)	−2.49 (−6.67, 1.69)
Other	63.2 (12.5)	−4.96 (−11.25, 1.33)	−4.72 (−10.58, 1.14)
Years in medical practice			
⩽10 years	65.1 (10.7)	0.00	0.00
11–20 years	67.7 (11.5)	2.66 (−0.80, 6.11)	3.03 (−1.24, 7.30)
21–30 years	67.1 (11.8)	1.92 (−1.52, 5.36)	1.78 (−2.79, 6.35)
>30 years	67.7 (13.5)	2.32 (−1.91, 6.54)	3.01 (−2.19, 8.21)
Actual hours per week in palliative medicine			
⩽20 h	68.4 (13.4)	0.28 (−5.59, 6.16)	0.62 (−5.07, 6.31)
21–30 h	63.4 (11.5)	**−4.32 (−7.44, −1.21)^d^**	**−3.35 (−6.34, −0.35)^d^**
31–40 h	67.8 (11.5)	0.00	0.00
41–50 h	67.0 (11.9)	−0.77 (−3.47, 1.94)	0.42 (−2.21, 3.06)
>50 h	70.7 (10.7)	2.70 (−0.80, 6.21)	**4.49 (1.13, 7.84)^d^**
Perceived level of support from others at clinical sites			
Spearman *r* (*p* value)	0.22	**1.25 (0.74, 1.77)^d^**	**1.23 (0.68, 1.78)^d^**
Patient Health Questionnaire-2 (PHQ-2)^b^			
<3	68.5 (11.1)	**−10.79 (−13.89, −7.79)^d^**	**−9.74 (−12.91, −6.58)^d^**
⩾3	57.6 (11.7)	0.00	0.00
Alcohol Use Disorders Identification Test – Consumption (AUDIT-C) score^c^			
0–3 (low-risk drinking)	67.4 (12.4)	0.00	0.00
4–5 (moderate-risk drinking)	67.3 (10.4)	−0.06 (−2.54, 2.42)	0.47 (−1.84, 2.78)
6–12 (high-risk drinking)	64.2 (11.4)	−3.09 (−6.60, 0.41)	−2.20 (−5.52, 1.13)

a Unless otherwise stated.

b Individuals with missing PHQ-2 scores excluded from analysis (*n* = 64, with 63 also missing CD-RISC score).

c Individuals with missing AUDIT-C scores excluded from analysis (*n* = 80, with 63 also missing CD-RISC score).

d Bold entries highlight the statistically significant results.

Bivariate associations between respondent characteristics (including variables that were not selected for the multivariable linear regression), burnout, and resilience are presented in Supplemental File 2.

## Discussion

To our knowledge, this is the first survey to specifically assess the prevalence of burnout and resilience amongst palliative medicine physicians in the UK and Ireland during the COVID-19 pandemic. There was a high response rate (67%). Our results demonstrate that a large proportion (39%) of palliative medicine physicians in the United Kingdom and Ireland are burnt out. We also discovered a strong association between higher burnout and lower resilience.

### Prevalence of burnout

Based on our primary study criteria for burnout (high emotional exhaustion or depersonalisation), 39.2% of physician respondents had burnout early in the pandemic. Comparable intrapandemic literature data are limited. In small subgroup surveys of Italian community-based palliative physicians in 2020 and 2021, 18.4%^44^ and 23.3%,^45^ respectively, had burnout based on slightly more restrictive MBI-HSS (MP) criteria (emotional exhaustion >27 or depersonalisation >10) than we used here. A pre-pandemic Italian survey, using the same criteria as in the 2020 and 2021 survey, reported a 44% prevalence of burnout in this same physician population.^44^ Other pre-pandemic literature reports variable levels of burnout using the MBI amongst palliative physicians in Canada (38%),^24^ Mexico (35%),^25^ Singapore (33%),^26^ United States (19%,^27^ 39%^28^) Spain (26%),^29^ Portugal (3%)^30–32^ and Japan (3%).^33^ Using the Copenhagen Burnout Inventory (CBI), a pre-pandemic Danish survey classified 20% of palliative physicians as having work-related burnout symptoms that required attention.^46^ More recently, an intrapandemic survey of Internal Medicine physicians in two Vancouver academic hospitals reported a 68% prevalence of burnout, using the same MBI criteria as in our survey.^47^ Our survey could have underestimated the prevalence of burnout, owing to a 33% non-response rate and the impact of our study’s definitional criteria for burnout. Using less restrictive criteria (high emotional exhaustion or depersonalisation, or low personal accomplishment) for burnout, gives a higher prevalence estimate of 47.4%, which is similar to the pre-pandemic, pooled 49% prevalence of burnout in unselected French physicians using the same criteria.^48^

### Contribution of the COVID-19 pandemic to burnout

Given the workforce burnout concerns raised by UK palliative care professionals during the pandemic,^49^ and those similarly reported by palliative care workers globally,^50^ the specific contribution of the pandemic to burnout prevalence, albeit difficult to quantify, warrants consideration. Palliative medicine providers in the UK have reported experiencing higher moral distress due to the pandemic preventing them from delivering care according to their professional values.^51,52^ Our survey was conducted during the pandemic, when palliative care services were often overwhelmed, yet felt ignored in the COVID-19 response at a health administrative level.^53^

### Degree of resilience

This study found a strong association between burnout and lower resilience, similar to a pre-pandemic Canadian palliative care physician survey.^24^ Although resilience scores were lower in Staff Grade and Associate Specialists, this respondent category was not associated with greater odds of burnout. Working over 50 h was associated with higher resilience, and although working less hours was associated with lower odds of burnout, it was also associated with less resilience. Less resilient physicians might also choose to work fewer hours. Greater perceived support from colleagues was associated with increased resilience, although physicians may bolster their resilience by actively seeking out support in times of need. Reporting depressive symptoms was associated with lower resilience, although it is unclear whether depression lowered an individual’s resilience or less resilient physicians were more susceptible to depression.

### Alcohol use

Similar to other physician reports,^54^ and as compared with their pre-pandemic consumption, 31.4% of our respondents reported increased alcohol intake during the pandemic. This may reflect a lack of other coping supports available to palliative medicine physicians at times when they are exposed to greater pressures or stresses in their clinical practice, as during the pandemic. Although alcohol consumption was not associated with burnout in our study, the association between alcohol intake and other health and social problems in physicians is well-described.^55^ Anxiety and hopelessness have been identified as common motivators for physicians to drink more alcohol.^54^

### Study implications and future research

There is a need to further investigate preventative factors and interventions for burnout. such investigations may help to identify methods for promoting physician well being and counteracting burnout or reducing burnout in those experiencing it. Exploring why the level of PA is so low in UK and Irish palliative medicine physicians compared with other groups,^24^ is needed to guide organisational change. Studies to date looking at interventions to decrease burnout in physicians have encouraged both system and individual level interventions.^56^ There is limited evidence to show whether or how physician resilience can be increased, but given its association with burnout, it merits further study.^57,58^ Working less than full time and greater perceived level of perceived colleague support were both protective against burnout in our survey. Both factors are strongly linked to the way healthcare services are organised and managed at a systems level and signal the need to target systemic causes of physician burnout in addressing this problem rather than focussing on individual-level interventions. Our finding of increased alcohol consumption in palliative medicine physicians is concerning and may be indicative of a maladaptive coping mechanism and could have longer term implications for physicians’ health and wellbeing as well as for clinical care. Future research should also examine the extent to which other factors, such as moral injury, have contributed to increased burnout during the COVID-19 pandemic, given that interventions to prevent or reduce such factors may need to be more specific.^59^ This may be particularly applicable to the speciality of palliative medicine.

### Strengths and limitations

In the absence of previously published data, our survey results, using validated tools, provide a benchmark for palliative medicine physicians in the UK and Ireland, which can be used as a baseline in assessing change, particularly organisational change. Our survey response rate (67%) was much higher than previous national surveys of palliative medicine physicians.^4,38,60,61^

The cross-sectional nature of the survey is a limitation. We cannot determine temporality between the associations observed in this study. Survey responses may have subsequently changed, and burnout may now be higher than earlier in the pandemic, given the cumulative stress associated with staffing and other demands arising in the pandemic’s subsequent waves. Also, although other studies suggest that burnout is higher in some palliative care settings,^62^ we were unable to further examine this, as most respondents work in a variety of care settings. There may be other predictors of burnout that were not assessed in this study. This study warrants careful comparison and interpretation in the context of burnout definitional criteria, survey response rates, different healthcare systems, the impact of the COVID-19, and other potential contributors.

## Conclusion

Over one-third of palliative medicine physicians meet the criteria for burnout. The prevalence of burnout and its associations, including low resilience, warrant further research and ongoing monitoring. Enhancing colleague support, backed up by organisations, is important to improve resilience and potentially decrease burnout in both the current and future pandemics.

## Supplemental Material

Supplementary File 1 Click here for additional data file. Survey information sheet and questions, excluding copyrighted instruments

Supplementary File 2 Click here for additional data file. Tables for bivariate associations between demographic variables, burnout, and resilience
