# Supplementary material for: Over a third of palliative medicine physicians meet burnout criteria: Results from a survey study during the COVID-19 pandemic
Source: Palliat Med. 2023 Feb 15;37(3):343–54. doi: 10.1177/02692163231153067 (PMC9936168; doi:10.1177/02692163231153067)
Supplement: Supplementary File 1 [file sj-pdf-1-pmj-10.1177_02692163231153067.pdf]

Supplementary File 1. Survey information sheet and questions, excluding copyrighted instruments.

Survey on Burnout and Resilience among Palliative Medicine Physicians in the UK and Ireland

Information sheet

Title of Study: Exploring burnout and resilience in UK and Irish Palliative Medicine Physicians
Local Site Principal Investigator (PI}: Dr. Jason Boland, Jason.Boland@hyms.ac.uk

Why was this e-mail sent to me?
You are being invited to take part in the above study as you are a member of the Association for Palliative Medicine (APM) of Great Britain and Ireland.

Why is this study being done?
This study is looking to assess the prevalence of burnout and degree of resilience in palliative medicine physicians across the UK and Ireland.

What will I need to do?
You will be asked to complete a series of questions regarding your burnout and resilience, all based on validated questionnaires. This survey will take approximately 10 minutes to complete.

Are there any risks involved with taking part in this study?
There is a low level of risk that you may not feel comfortable with all of the questions that are asked. You can stop the survey at any time if you feel uncomfortable. If you experience any feelings of distress during the completion of this survey, please seek support as appropriate, including from your general practitioner if needed.

Can I expect to benefit from participating?
You are unlikely to benefit directly from being a part of this study. However, taking part in this study will help the research team to understand and disseminate insights into burnout and resilience in palliative medicine physicians.

Will I be paid or have to pay to take part in this study?
You will not be paid or have to cover any costs for taking part in this study.

How will my personal information be protected?
Any survey data that will be collected as part of this study is anonymous, and therefore will not contain information that identifies you.

Do I have to participate? What options do I have?
Your participation in this study is voluntary. You may choose not to take part at all. Although answers to all questions are mandatory in order to allow your responses to be submitted for analysis, you may stop taking part at any time by exiting the survey, in which case your incomplete survey responses will not be submitted for analysis. As survey data is anonymous, if you indicate that you wish to withdraw after completing the survey, your submitted survey data cannot be withdrawn as there are no links between participants and the survey.

Conflict of Interest
There are no conflicts of interest on the part of the researchers or their institutions.

Will I be informed about the study results?
We expect to have results of the study by August 2021. Results will be disseminated via the APM.

What do I do if I have questions about the study?
The research team would be happy to address any questions you might have at any time. If you have any questions, you can contact Dr. Jason Boland, the Principal Investigator at Jason.Boland@hyms.ac.uk

For questions or complaints related to ethical conduct of the study, you may contact: The Hull York Medical School Ethics Committee (ethics@hyms.ac.uk).

The Hull York Medical School Ethics Committee has reviewed and approved the ethical conduct of the study.

You may save or print a screenshot of this information sheet before taking part in this study.

Survey on Burnout and Resilience among Palliative Medicine Physicians in the UK and Ireland

Consent Form

Consent to Participate in Research:

- **I understand that I am being asked to participate in a research study on UK and Irish palliative medicine physicians' experiences of burnout and resilience.**
- **I have read the information sheet and all of my questions have been answered to my satisfaction.**
- **If I decide that I would like to withdraw my participation and/or consent from the study, I can do so at any time during the survey. I understand that submitted survey data cannot be withdrawn as there are no links between the participant and the survey.**
- **I voluntarily agree to participate in this study. I can save or print a screenshot of this completed Consent Form for my own record.**

* 1. In checking the box below I voluntarily agree to participate in this study

◯ I agree

Survey on Burnout and Resilience among Palliative Medicine Physicians in the UK and Ireland

Demographics

* 2. What age category are you in?

◯ 21-30
◯ 31-40
◯ 41-50
◯ 51-60
◯ 61-65
◯ >65

*** 3. What is your gender?**

- Male
- Female
- Prefer not to answer
- Other (please specify)

*** 4. I am a:**

- Consultant in Palliative Medicine
- Staff grade or Associate Specialist in Palliative Medicine
- Specialist Trainee or Registrar in Palliative Medicine
- Other (please specify)

*** 5. How long have you been in medical practice?**

- <5 years
- 5-10 years
- 11-20 years
- 21-30 years
- >30 years

*** 6. How long have you been working in palliative medicine?**

- Have not worked in palliative medicine
- <1 year
- 1-4 years
- 5-9 years
- 10-20 years
- >20 years

*** 7. I provide palliative medicine in (select all that apply):**

- [ ] Cancer centre
- [ ] Community Hospital
- [ ] General Hospital
- [ ] Teaching Hospital
- [ ] Hospice Inpatient Unit
- [ ] Long Term Care / Nursing or Care Home
- [ ] Patient's Home
- [ ] Outpatient Clinic Care
- [ ] Other (please specify)

*** 8. According to your contract, how many hours per week, excluding on-call, are you required to work in palliative medicine (i.e. clinical, teaching, research, management and administrative tasks)?**

- ○ 0-10 hours
- ○ 11-20 hours
- ○ 21-30 hours
- ○ 31-40 hours
- ○ 41-50 hours
- ○ 51-60 hours
- ○ >60 hours

*** 9. In a typical week, how many hours in total do you actually work per week in palliative medicine (including clinical, teaching, research, management and administrative tasks)?**

- ○ 0-10 hours
- ○ 11-20 hours
- ○ 21-30 hours
- ○ 31-40 hours
- ○ 41-50 hours
- ○ 51-60
- ○ >60 hours

*** 10. Do you provide on-call (outside of regular working hours) for palliative medicine?**

- ○ Yes
- ○ No

Demographics

*** 11. How often do you provide on-call (outside of regular working hours) for palliative medicine? Please select the nearest approximation.**

- 1 in 7 or less frequently
- 1 in 4 to 1 in 6
- 1 in 3 or more frequently

Survey on Burnout and Resilience among Palliative Medicine Physicians in the UK and Ireland

Demographics

*** 12. Do you receive formal (professional) supervision for your work in palliative medicine?**

- Yes
- No

*** 13. How well supported by others do you feel at your practice site(s)?**

0 (Not at all) 5 10 (Very much)

*** 14. Over the past 12 months, in a typical month: Of your time spent working, roughly what percentage did you spend in each of the following? Please provide a percentage figure without the percentage (%) sign. The total should amount to 100.**

Palliative medicine clinical work

Non-palliative medicine clinical work

Non-clinical work (education, research, administrative)

Other work (specify in next question)

15. If you spent time doing "other work" in question above, please specify:

Survey on Burnout and Resilience among Palliative Medicine Physicians in the UK and Ireland

*** 25. Over the past two weeks, how often have you been bothered by any of the following problems?** [1]

	Not at all	Several days	More than half the days	Nearly every day
Little interest or pleasure in doing things.	◯	◯	◯	◯
Feeling down, depressed, or hopeless.	◯	◯	◯	◯

[1] Reference listed at end of survey.

Survey on Burnout and Resilience among Palliative Medicine Physicians in the UK and Ireland

*** 26. How often do you have a drink* containing alcohol?** [2,3]

Never	Monthly or less	Two to four times a month	Two to three times a week	Four or more times a week
◯	◯	◯	◯	◯

27. How many drinks* containing alcohol do you have on a typical day when you are drinking? [2,3]

1 or 2	3 or 4	5 or 6	7 to 9	10 or more
◯	◯	◯	◯	◯

*** 28. How often do you have six or more drinks* on one occasion?** [2,3]

Never	Less than monthly	Monthly	Weekly	Daily or almost daily
◯	◯	◯	◯	◯

*** 29. Do you think your alcohol intake has changed since the start of COVID-19?**

- ◯ Decreased a lot
- ◯ Decreased a little
- ◯ Stayed about the same
- ◯ Increased a little
- ◯ Increased a lot

*** 1 drink = 1 unit**

[2,3] References listed at end of survey.

Survey on Burnout and Resilience among Palliative Medicine Physicians in the UK and Ireland

*** 30. How would you rate your overall well-being over the past week?** [4]

0 (As bad as it can be) | 5 | 10 (As good as it can be)

*** 31. In a typical week, how overwhelmed do you feel at work?**

0 (Not at all) | 5 | 10 (Very much)

32. What are the benefits of working in palliative medicine in relation to your mental and physical health and job satisfaction?

33. What are the major challenges of working in palliative medicine in relation to your mental and physical health and job satisfaction?

[4] Reference listed at end of survey.

Survey on Burnout and Resilience among Palliative Medicine Physicians in the UK and Ireland

34. From what or where do you find support to help you minimize stress, and get the most out of your work?

35. Is there anything else that you would like to share regarding burnout, resilience and well-being?

36. Any other comments?

Survey on Burnout and Resilience among Palliative Medicine Physicians in the UK and Ireland

Thank you for completing this survey.

If you experienced distress during the completion of this survey, please seek support as appropriate, including from your general practitioner if needed.

References

1. Kroenke K, Spitzer RL, Williams JB. The Patient Health Questionnaire-2: validity of a two-item depression screener. Med Care 2003;41(11):1284-1292. doi:10.1097/01.MLR.0000093487.78664.3C
2. Bush K, Kivlahan DR, McDonell MB, et al. The AUDIT alcohol consumption questions (AUDIT-C): an effective brief screening test for problem drinking. Ambulatory Care Quality Improvement Project (ACQUIP). Alcohol Use Disorders Identification Test. Arch Intern Med 1998;158(16):1789-1795. doi:10.1001/archinte.158.16.1789
3. Bradley KA, Bush KR, Epler AJ, et al. Two brief alcohol-screening tests From the Alcohol Use Disorders Identification Test (AUDIT): validation in a female Veterans Affairs patient population. Arch Intern Med 2003;163(7):821-829. doi:10.1001/archinte.163.7.821
4. Locke DEC, Decker PA, Sloan JA, et al. Validation of Single-Item Linear Analog Scale Assessment of Quality of Life in Neuro-Oncology Patients. J Pain Symptom Manage 2007; 34: 628-638. DOI: https://doi.org/10.1016/j.jpainsymman.2007.01.016.
