# Supplementary material for: Over a third of palliative medicine physicians meet burnout criteria: Results from a survey study during the COVID-19 pandemic
Source: Palliat Med. 2023 Feb 15;37(3):343–54. doi: 10.1177/02692163231153067 (PMC9936168; doi:10.1177/02692163231153067)
Supplement: Supplementary File 2 [file sj-pdf-2-pmj-10.1177_02692163231153067.pdf]

Supplementary File 2. Tables for bivariate associations between respondent characteristics, burnout and resilience.

Supplementary Table 1. Bivariate associations between respondent characteristics and burnout, excluding individuals with missing MBI-HSS (MP) data for outcome measurement (row %).

	Burnout n (%)	No burnout n (%)	p
Age			0.03
21-30	7 (43.7)	9 (56.2)	
31-40	45 (31.7)	97 (68.3)	
41-50	76 (40.2)	113 (59.8)	
51-60	52 (49.5)	53 (50.5)	
61-65	1 (12.5)	7 (87.5)	
>65	0 (0.0)	2 (100.0)	
Gender			0.42
Male	36 (43.9)	46 (56.1)	
Female	145 (38.4)	233 (61.6)	
Prefer not to answer	0 (0.0)	2 (100.0)	
Role			0.01
Consultant in Palliative Medicine	133 (44.6)	165 (55.4)	
Staff grade or Associate Specialist in Palliative Medicine	25 (31.6)	54 (68.3)	
Specialist Trainee or Registrar in Palliative Medicine	18 (25.3)	53 (74.6)	
Other	5 (35.7)	9 (64.3)	
Years in medical practice			0.01
<5 years	2 (66.7)	1 (33.3)	
5 to 10 years	13 (21.7)	47 (78.3)	
11 to 20 years	62 (36.7)	107 (63.3)	
21-30 years	75 (43.9)	96 (56.1)	
>30 years	29 (49.1)	30 (50.8)	
Years in Palliative Medicine			0.07
<1 year	2 (22.2)	7 (78.8)	
1-4 years	16 (28.6)	40 (71.4)	
5-9 years	33 (32.3)	69 (67.6)	
10-20 years	87 (44.6)	108 (55.4)	
>20 years	43 (43.0)	57 (57.0)	
Contracted hours per week in Palliative Medicine			0.07
0-10 hours	2 (28.6)	5 (71.4)	
11-20 hours	4 (14.8)	23 (85.2)	
21-30 hours	51 (37.8)	84 (62.2)	
31-40 hours	97 (41.8)	135 (58.2)	
41-50 hours	25 (43.1)	33 (56.9)	
51-60 hours	2 (66.7)	1 (33.3)	
Actual hours per week in Palliative Medicine			0.002
0-10 hours	2 (66.7)	1 (33.3)	

11-20 hours	0 (0.0)	16 (100.0)	
21-30 hours	28 (31.8)	60 (68.2)	
31-40 hours	51 (35.2)	94 (64.8)	
41-50 hours	69 (46.9)	78 (53.1)	
51-60 hours	26 (52.0)	24 (48.0)	
>60 hours	5 (38.5)	8 (61.5)	
Difference between contracted and actual hours			0.01
0 hours (i.e. contracted was the same or less than actual)	67 (32.1)	142 (67.9)	
1-10 hours more	90 (43.3)	118 (56.7)	
11-20 hours more	21 (56.8)	16 (43.2)	
21-30 more	3 (37.5)	5 (62.5)	
Provision of on-call for Palliative Medicine			0.32
Yes	162 (38.5)	259 (61.5)	
No	19 (46.3)	22 (53.7)	
Frequency of on-call for Palliative Medicine			0.11
1 in 7 or less frequently	56 (41.8)	78 (58.2)	
1 in 4 to 1 in 6	99 (38.8)	156 (61.2)	
1 in 3 or more frequently	7 (21.9)	25 (78.1)	
Formal supervision in Palliative Medicine			<0.001
Yes	56 (29.6)	133 (70.4)	
No	125 (45.8)	148 (54.2)	
Level of support from others at practice sites			
Median (IQR)	7 (5-8)	8 (7-9)	<0.001
Percent time spent in: (n responded)			
Palliative Medicine clinical work (n=489)			
Non-Palliative Medicine clinical work (n=288)			
Non-clinical work (n=477)			
Other work (n=145)			
Patient Health Questionnaire-2 (PHQ-2)[a]			<0.001
0	24 (12.6)	167 (87.5)	
1	29 (37.7)	48 (62.3)	
2	71 (56.3)	55 (43.6)	
3	19 (86.4)	3 (13.6)	
4	23 (92.0)	2 (8.0)	
5	3 (100.0)	0 (0.0)	
6	7 (87.5)	1 (12.5)	
Alcohol Use Disorders Identification Test – Consumption			0.049
(AUDIT-C) score[b]	88 (34.8)	165 (65.2)	
0-3 (low-risk drinking)	55 (41.9)	76 (58.0)	
4-5 (moderate-risk drinking)	27 (51.9)	25 (48.1)	
6-12 (high-risk drinking)			

[a] Individuals with missing PHQ-2 scores were excluded from analysis (n=64, with 54 also missing burnout score)

[b] Individuals with missing AUDIT-C scores were excluded from analysis (n=80, with 54 also missing burnout score)

Supplementary Table 2. Bivariate associations between respondent characteristics and mean Connor-Davidson Resilience Scale (CD-RISC) scores, excluding individuals with missing CD-RISC data for outcome measurement

	Mean score (SD)	p
Age		0.57
21-30	64.1 (7.9)	
31-40	66.2 (10.6)	
41-50	67.9 (12.6)	
51-60	67.0 (12.2)	
61-65	67.9 (14.7)	
>65	76.0 (5.6)	
Gender		0.34
Male	66.6 (11.9)	
Female	67.1 (11.8)	
Prefer not to answer	79.0 (5.6)	
Role		0.03
Consultant in Palliative Medicine	68.2 (11.9)	
Staff grade or Associate Specialist in Palliative Medicine	64.3 (12.0)	
Specialist Trainee or Registrar in Palliative Medicine	66.4 (10.2)	
Other	63.2 (12.5)	
Years in medical practice		0.57
<5 years	60.3 (9.3)	
5 to 10 years	65.3 (10.8)	
11 to 20 years	67.7 (11.5)	
21-30 years	67.1 (11.8)	
>30 years	67.7 (13.5)	
Years in Palliative Medicine		0.09
<1 year	67.3 (5.7)	
1-4 years	63.6 (10.6)	
5-9 years	66.1 (10.8)	
10-20 years	68.0 (12.3)	
>20 years	68.4 (12.4)	
Contracted hours per week in Palliative Medicine		0.002
0-10 hours	75.8 (12.5)	
11-20 hours	64.6 (10.5)	
21-30 hours	64.3 (11.7)	
31-40 hours	68.2 (11.7)	
41-50 hours	69.8 (11.1)	
51-60 hours	58.3 (17.4)	
Actual hours per week in Palliative Medicine		0.01
0-10 hours	65.0 (9.9)	
11-20 hours	68.9 (14.0)	
21-30 hours	63.4 (11.5)	
31-40 hours	67.8 (11.5)	
41-50 hours	67.0 (11.9)	
51-60 hours	69.5 (11.2)	
>60 hours	75.1 (7.6)	

Difference between contracted and actual hours		0.29
0 hours (i.e. contracted was the same or less than actual)	66.4 (11.9)	
1-10 hours more	67.3 (11.8)	
11-20 hours more	69.0 (11.2)	
21-30 more	73.0 (6.2)	
Provision of on-call for Palliative Medicine		0.95
Yes	67.1 (11.7)	
No	67.2 (12.9)	
Frequency of on-call for Palliative Medicine		0.09
1 in 7 or less frequently	65.6 (11.3)	
1 in 4 to 1 in 6	67.5 (11.6)	
1 in 3 or more frequently	70.4 (13.1)	
Formal supervision in Palliative Medicine		0.98
Yes	67.1 (10.9)	
No	67.1 (12.3)	
Level of support from others at practice sites	0.22	<0.001
Spearman r (p value)		
Percent time spent in: (n responded)		
Palliative Medicine clinical work (n=489)		
Non-Palliative Medicine clinical work (n=288)		
Non-clinical work (n=477)		
Other work (n=145)		
Patient Health Questionnaire-2 (PHQ-2)[a]		<0.001
0	72.9 (10.2)	
1	66.4 (10.0)	
2	63.1 (10.6)	
3	57.1 (9.6)	
4	59.1 (12.7)	
5	63.0 (6.5)	
6	52.6 (15.3)	
Alcohol Use Disorders Identification Test – Consumption (AUDIT-C) score[b]	67.4 (12.4)	0.20
0-3 (low-risk drinking)	67.3 (10.4)	
4-5 (moderate-risk drinking)	64.2 (11.4)	
6-12 (high-risk drinking)		

[a] Individuals with missing PHQ-2 scores were excluded from analysis (n=64, with 63 also missing CD-RISC score)

[b] Individuals with missing AUDIT-C scores were excluded from analysis (n=80, with 63 also missing CD-RISC score)
